# Short communication: Estimation of the dietary standardized ileal digestible valine to lysine ratio required for 40 to 130 kg pigs during the finisher periods

**DOI:** 10.1186/s40813-024-00405-6

**Published:** 2024-11-07

**Authors:** Zijuan Wu, Wenli Li, Yali Li

**Affiliations:** Hunan Provincial Key Laboratory of Animal Intestinal Function and Regulation, Hunan Normal University, No.36 Lushan Road, Changsha, 410081 Hunan China

**Keywords:** Val, Amino acid ratio, Requirement, Growth performance, Finishing pigs

## Abstract

**Background:**

Three experiments were conducted separately to determine the optimum standardized ileal digestible (SID) valine (Val) to lysine (Lys) ratio for early finishing (Experiment 1, 40 to 75 kg bodyweight), finishing (Experiment 2, 75 to 100 kg bodyweight), and late finishing (Experiment 3, 100 to 130 kg bodyweight) pigs. Dietary SID Val: Lys ratios were designed at 0.61, 0.65, 0.69, 0.73, and 0.77. The optimal SID Val: Lys ratio was estimated by different regression models, including a quadratic polynomial model, a two-slope quadratic broken-line model, a curvilinear-plateau model, and a one-slope straight broken-line model.

**Results:**

In Exp.1, a total of 550 early finishing pigs (initially 40.3 kg bodyweight) were used in a 38-day growth trial. Pigs consuming 0.61 dietary SID Val: Lys ratio had lower final bodyweight compared to those fed 0.69 in diets. Using regression models, the optimal dietary SID Val: Lys requirement for average daily gain (ADG) was between 0.63 and 0.68, and for feed to gain ratio (F: G) was between 0.62 and 0.68, respectively. In Exp.2, 525 finishing pigs (initially 76.4 kg) were used in a 26-day trial. Based on regression models, estimate of the required SID Val: Lys for ADG was between 0.65 and 0.71, and for F: G was between 0.64 and 0.70, respectively. In Exp.3, 640 late finishing pigs (102 kg bodyweight) were used in a 27-day trial. No significant improvement was found for performance parameters of pigs from 100 to 130 kg, while 0.73 SID Val: Lys ratio resulted in the highest ADG and the lowest F: G from a numerical point of view.

**Conclusions:**

These findings indicated that the optimum SID Val: Lys requirement for pigs from 40 to 75 kg was between 0.62 and 0.68, and for pigs from 75 to 100 kg was estimated to be between 0.64 and 0.71, using different regression models.

## Background

Valine (Val) is generally considered to be the fifth limiting amino acid (AA) after lysine (Lys), methionine (Met), threonine (Thr), and tryptophan (Trp) in corn-soybean meal-based diets for pigs [1, 2]. Val is a branched-chain essential amino acid (BCAA) and plays critical roles in appetite regulation, muscle protein synthesis, and insulin secretion [3, 4]. Deficiency of Val may lead to a depression in feed intake and consequently to poor growth performance in pigs [3, 5]. Thus, it is necessary to ensure appropriate levels of Val in swine diets, for better economic and environmental outcomes. However, few empirical studies have been devoted to investigate the Val requirement for finishing pigs [6].

An understanding of the nutrient requirements across various conditions was crucial for the decision of the most economical method of feeding pigs [7]. Many factors, like breed, production stage, or housing environment may affect the requirements [5]. Recently, the use of antibiotics in animal feeds has been banned in China, and therefore the optimal supplemental amount of AA for maximal performance and economic return may need to be re-estimated under antibiotic-free conditions [8]. The ideal AA profile is often expressed relative to Lys. Therefore, this research was conducted to estimate the optimum SID Val: Lys ratio for best growth performance and feed efficiency for pigs fed corn-soybean meal diets using regression approaches. Given that the ideal ratio of Val to Lys varies with different production stages, three experiments were performed to evaluate optimal Val requirements for early finishing (Exp.1, 40 to 75 kg bodyweight), finishing (Exp.2, 75 to 100 kg bodyweight), and late finishing (Exp.3, 100 to 130 kg bodyweight) pigs under commercial conditions.

## Methods

### Experimental design and diets

All animal procedures used in this study were approved by the Institutional Animal Care and Use Committee of Hunan Normal University (2019 − 170). Experimental pigs [(Landrace × Yorkshire) × Duroc] were housed in pens at a commercial research facility (Anyuan, Jiangxi, China). Similar number of barrows and gilts were placed in each pen. Feed and water were provided *ad libitum*. The SID Lys was formulated 0.10% points below the levels recommended by NRC (2012) [9] to ensure that pigs were below the Lys requirement. A basal SID Val: Lys ratio of 0.61 was used in the current study. SID concentrations of AA were estimated using SID coefficients for various ingredients as provided by NRC (2012) [9]. All other nutrient requirements met the NRC (2012) [9] recommendations.

Experiment 1 (Exp.1) was conducted to explore the optimal dietary SID Val: Lys ratio for early finishing pigs (40 to 75 kg bodyweight). A total of 550 pigs, with an initial bodyweight of 40.3 ± 0.8 kg, were randomly allotted to 5 dietary treatments. Graded amounts of crystalline L-Val (0, 0.30, 0.61, 0.91, or 1.21 g kg^− 1^ diet; Meihua Group, Hebei, China) were supplemented to obtain SID Val: Lys ratios of 0.61, 0.65, 0.69, 0.73, and 0.77. The SID Lys level for Exp.1 was set at 7.50 g kg^− 1^ diet (second limiting), and the crude protein (CP) content was set at 128 g kg^− 1^ diet. Each treatment group had 5 replicate pens with 22 pigs per pen. The experiment lasted 38 days.

In Exp.2, 525 finishing pigs (initially 76.4 ± 0.97 kg bodyweight) were randomly allocated to 5 dietary treatments and experiment lasted 26 days. L-Val (0, 0.25, 0.51, 0.76, or 1.02 g kg^− 1^ diet) were added to produce diets providing SID Val: Lys ratios of 0.61, 0.65, 0.69, 0.73, and 0.77. The SID Lys level for Exp.2 was set at 6.30 g kg^− 1^ diet (second limiting), and the CP was 109 g kg^− 1^ diet. Each treatment had 5 replicates with 21 pigs per replication.

In Exp.3, 640 late finishing pigs, with an average initial bodyweight of 102 ± 1.80 kg, were randomly assigned to 5 dietary treatments. Diets were formulated to contain 0, 0.21, 0.41, 0.62, or 0.82 g kg^− 1^ diet of supplemental Val to provide SID Val: Lys ratios of 0.61, 0.65, 0.69, 0.73, and 0.77. The SID Lys level was set at 5.10 g kg^− 1^ diet (second limiting), and the CP was 88.7 g kg^− 1^ diet. Each treatment had 4 replicates with 32 pigs per replication. The feeding trial was conducted for 27 days.

To avoid potential compensatory growth, the finishing pigs used in Exp.1, 2 and 3 were obtained from three different batches. Experimental diets were based on corn, soybean meal, wheat bran, or corn starch, supplemented with crystalline AA. Diet samples were analyzed for crude nutrient content using AOAC (2012) methods [10]. CP content of feeds was calculated as Kjeldahl-*N* × 6.25. AA composition was determined by a L-8900 Amino Acid Analyzer (HITACHI, Tokyo, Japan). Ingredient and nutrient composition of experimental diets are presented in Table 1.

**Table 1:** Ingredient, calculated and analyzed nutrient composition of the diets (g kg^− 1^ as-fed)

Item	Exp.1(40–75 kg)		Exp.2(75–100 kg)	Exp.3(100–130 kg)
Ingredients				
Corn	797		823	786
Soybean meal (43.9% CP)	111		48.1	21.2
Wheat bran	56.1		95.9	61.2
Corn Starch	0		0	100
Limestone	7.66		7.74	6.84
Dicalcium phosphate	11.3		8.37	8.22
Salt	4.00		4.00	4.00
Choline chloride	0.70		0.70	0.70
Vitamin-mineral premix^†^	5.00		5.00	5.00
L-Lys sulfate 70.0%	5.63		5.87	5.24
L-Thr 98.5%	1.11		1.14	1.14
DL-Met 99.0%	0.24		0	0
L-Trp 98.0%	0.29	0.34		0.34
L-Val 99.0%^‡^	Variable		Variable	Variable
L-Ile 99.0%	0.03		0.29	0.37
Calculated composition				
CP	128		109	88.7
NE, MJ	10.4		10.4	10.4
SID-Lys	7.50		6.30	5.10
SID-(Met + Cys): Lys	0.57		0.59	0.60
SID-Thr: Lys	0.62		0.63	0.66
SID-Trp: Lys	0.18		0.18	0.18
SID-Val: Lys	0.61		0.61	0.61
SID-Ile: Lys	0.53		0.53	0.54
SID-Leu: Lys	1.37		1.40	1.48
SID-His: Lys	0.39		0.39	0.39
Analyzed composition^§^				
Dry matter	881		872	871
CP	132		114	92.8
Total Lys	8.25		6.93	5.61
Total Met + Cys	4.95		4.42	4.24
Total Thr	5.49		4.68	3.92
Total Trp	1.71		1.51	1.35
Total Val	5.32		4.46	3.64
Total Ile	5.33		5.07	3.78
Total Leu	11.55		8.85	8.53

Abbreviations: SID = standardized ileal digestible; CP = crude protein; NE = net energy

^†^Premix provided the following per kilogram of complete diet in Exp.1: vitamin A, 9,000 IU; vitamin D_3_, 2,400 IU; vitamin E, 20 IU; vitamin K_3_, 3 mg; thiamine, 1.4 mg; riboflavin, 4 mg; pyridoxine, 3 mg; vitamin B12, 12 µg; nicotinic acid, 30 mg; pantothenic acid, 14 mg; folic acid, 0.8 mg; biotin, 44 µg; Fe, 76 mg; Cu, 240 mg; Zn, 76 mg; Mn, 20 mg; I, 0.48 mg; Se, 0.4 mg; Premix provided the following per kilogram of complete diet in Exp.2 and Exp.3: vitamin A, 6,000 IU; vitamin D_3_, 2,400 IU; vitamin E, 20 IU; vitamin K_3_, 2 mg; thiamine, 0.96 mg; riboflavin, 5.3 mg; pyridoxine, 2 mg; vitamin B12, 12 µg; nicotinic acid, 22 mg; pantothenic acid, 11.2 mg; folic acid, 0.4 mg; biotin, 40 µg; Fe, 76 mg; Cu, 120 mg; Zn, 76 mg; Mn, 12 mg; I, 0.24 mg; Se, 0.4 mg

^‡^L-valine added at 0, 0.30, 0.61, 0.91, and 1.21 g kg^− 1^ to the basal diet in Exp.1; L-valine added at 0, 0.25, 0.51, 0.76, and 1.02 g kg^− 1^ to the basal diet in Exp.2; L-valine added at 0, 0.21, 0.41, 0.62, and 0.82 g kg^− 1^ to the basal diet in Exp.3

^§^Three samples of each diet were analyzed (*n* = 3)

### Data collection and statistical analysis

Pigs per pen were weighed and feed disappearance was recorded to determine average daily feed intake (ADFI), average daily gain (ADG), and feed to gain ratio (F: G). Data were analyzed by one-way ANOVA followed by Tukey’s *post hoc* test. Polynomial contrasts were conducted to examine linear and quadratic effects of increasing SID Val: Lys ratios. *P*-value < 0.05 was considered significant. The optimal SID Val: Lys ratios were estimated by different regression models (including a quadratic polynomial model, a two-slope quadratic broken-line model, a curvilinear-plateau model, and a straight broken-line model), using the NLIN procedure of SAS (SAS Inst. Inc., Cary, NC) [8, 11].

## Results

### Experiment 1

As shown in Table 2, early finishing pigs that were fed 0.61 dietary SID Val: Lys ratio had lower final bodyweight (FBW) compared to those fed 0.69 and the FBW was found to be improved in a quadratic manner with SID Val: Lys ratio increasing (*P* < 0.05). ADFI was not affected by Val addition while daily SID Val intake and Val: G (SID Val intake to ADG ratio) significantly increased with increasing Val supplementation levels.

**Table 2:** Performance of pigs during early finishing periods (experiment 1)

	SID Val to Lys ratio						*P* value		
Item	0.61	0.65	0.69	0.73	0.77	SEM	ANOVA	Linear	Quadratic
IBW, kg	39.8	39.8	40.5	40.7	40.6	0.16	0.239	0.044	0.550
FBW, kg	74.7^b^	75.8^ab^	76.7^a^	76.0^ab^	75.1^ab^	0.22	0.023	0.414	0.002
ADFI, kg	2.31	2.26	2.32	2.30	2.31	0.03	0.601	0.694	0.618
Val intake, g	10.6^d^	11.1^d^	12.0^c^	12.6^b^	13.7^a^	0.23	0.000	0.000	0.090
Val:G	11.5^d^	11.7^d^	12.6^c^	13.6^b^	15.1^a^	0.27	0.000	0.000	0.000
ADG, kg	0.92^b^	0.95^a^	0.95^a^	0.93^ab^	0.91^b^	0.01	0.001	0.140	0.000
F:G	2.52^a^	2.39^b^	2.43^ab^	2.47^ab^	2.54^a^	0.02	0.015	0.214	0.003

Abbreviations: SID = standardized ileal digestible; IBW = initial bodyweight; FBW = final bodyweight; ADFI = average daily feed intake; Val intake = daily SID Val intake; Val: G = daily SID Val intake (g)/average daily gain (kg); ADG = average daily gain; F:G = feed to gain ratio; *n* = 5

**Fig. 1:**
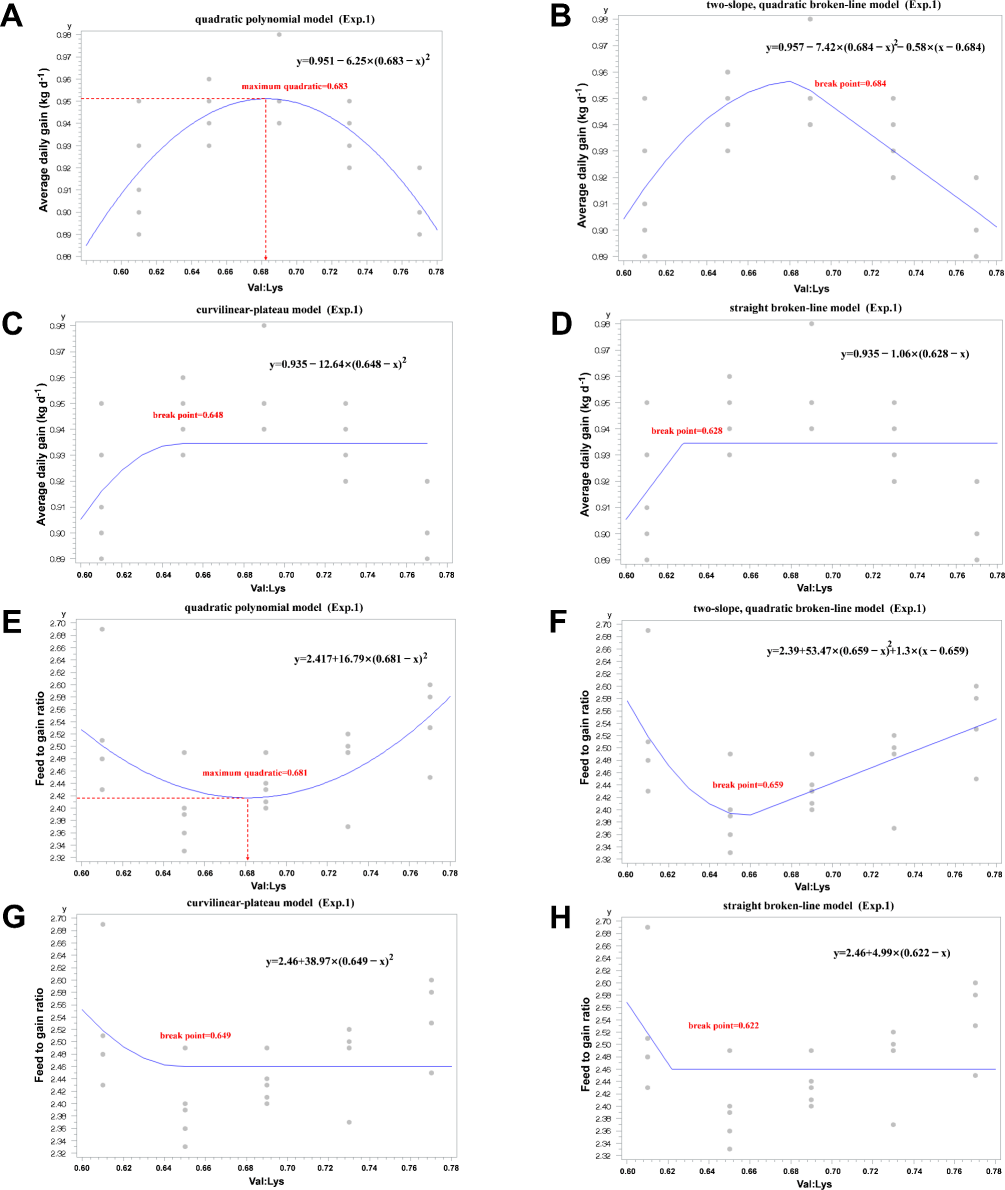
Estimation of dietary SID Val: Lys ratio for early finishing pigs (Exp.1). Fitted plot (quadratic polynomial model, two-slope quadratic broken-line model, curvilinear-plateau model, and straight broken-line model) of ADG (**A-D**) and **F**: **G** (**E-H**) for pigs (40 to 75 kg bodyweight). The optimal Val requirement for ADG was 0.683, 0.684, 0.648, and 0.628, respectively (**A-D**). The optimal Val requirement for **F**: **G** was 0.681, 0.659, 0.649, and 0.622, respectively (**E-H**)

Both ADG and F: G showed significant quadratic (*P* < 0.05) response with an increasing dietary SID Val: Lys ratio. Using regression models, the optimum dietary SID Val: Lys requirement to maximize ADG for 40 to 75 kg early finishing pigs were 0.68 (based on a quadratic polynomial model [R^2^ = 0.57] and a two-slope quadratic broken-line model [R^2^ = 0.59]), 0.65 (curvilinear-plateau model [R^2^ = 0.10]), and 0.63 (straight broken-line model [R^2^ = 0.11]), respectively (Fig. 1A, B, C, and D). Moreover, the optimal Val requirement for F: G was 0.68 (quadratic polynomial model [R^2^ = 0.58]), 0.66 (two-slope quadratic broken-line model [R^2^ = 0.79]), 0.65 (curvilinear-plateau model [R^2^ = 0.09]), and 0.62 (straight broken-line model [R^2^ = 0.09]), respectively (Fig. 1E, F, G, and H).

### Experiment 2

As presented in Table 3, finishing pigs receiving 0.61 dietary SID Val: Lys ratio had lower ADFI compared to those fed 0.69 or 0.73 SID Val: Lys in diets (*P* < 0.05). Notably, there was a quadratic improvement in FBW (*P* < 0.05) while both linear and quadratic increases were observed in ADFI, Val intake, and Val: G as the SID Val: Lys ratio increased in diets (*P* < 0.05).

**Table 3:** Performance of pigs during finishing periods (experiment 2)

	SID Val to Lys ratio						*P* value		
Item	0.61	0.65	0.69	0.73	0.77	SEM	ANOVA	Linear	Quadratic
IBW, kg	76.8	77.2	75.7	76.2	76.1	0.19	0.093	0.068	0.527
FBW, kg	100	103	104	103	102	0.52	0.089	0.422	0.009
ADFI, kg	2.91^b^	3.07^ab^	3.17^a^	3.15^a^	3.06^ab^	0.03	0.005	0.016	0.001
Val intake, g	11.1^d^	12.4^c^	13.7^b^	14.4^b^	15.1^a^	0.30	0.000	0.000	0.021
Val:G	12.2^c^	12.4^bc^	12.4^c^	13.9^b^	15.4^a^	0.29	0.000	0.000	0.004
ADG, kg	0.91^b^	1.00^ab^	1.11^a^	1.04^ab^	0.99^ab^	0.02	0.013	0.107	0.002
F:G	3.22	3.07	2.87	3.04	3.11	0.04	0.062	0.343	0.011

Abbreviations: SID = standardized ileal digestible; IBW = initial bodyweight; FBW = final bodyweight; ADFI = average daily feed intake; Val intake = daily SID Val intake; Val: G = daily SID Val intake (g)/average daily gain (kg); ADG = average daily gain; F:G = feed to gain ratio; *n* = 5

**Fig. 2:**
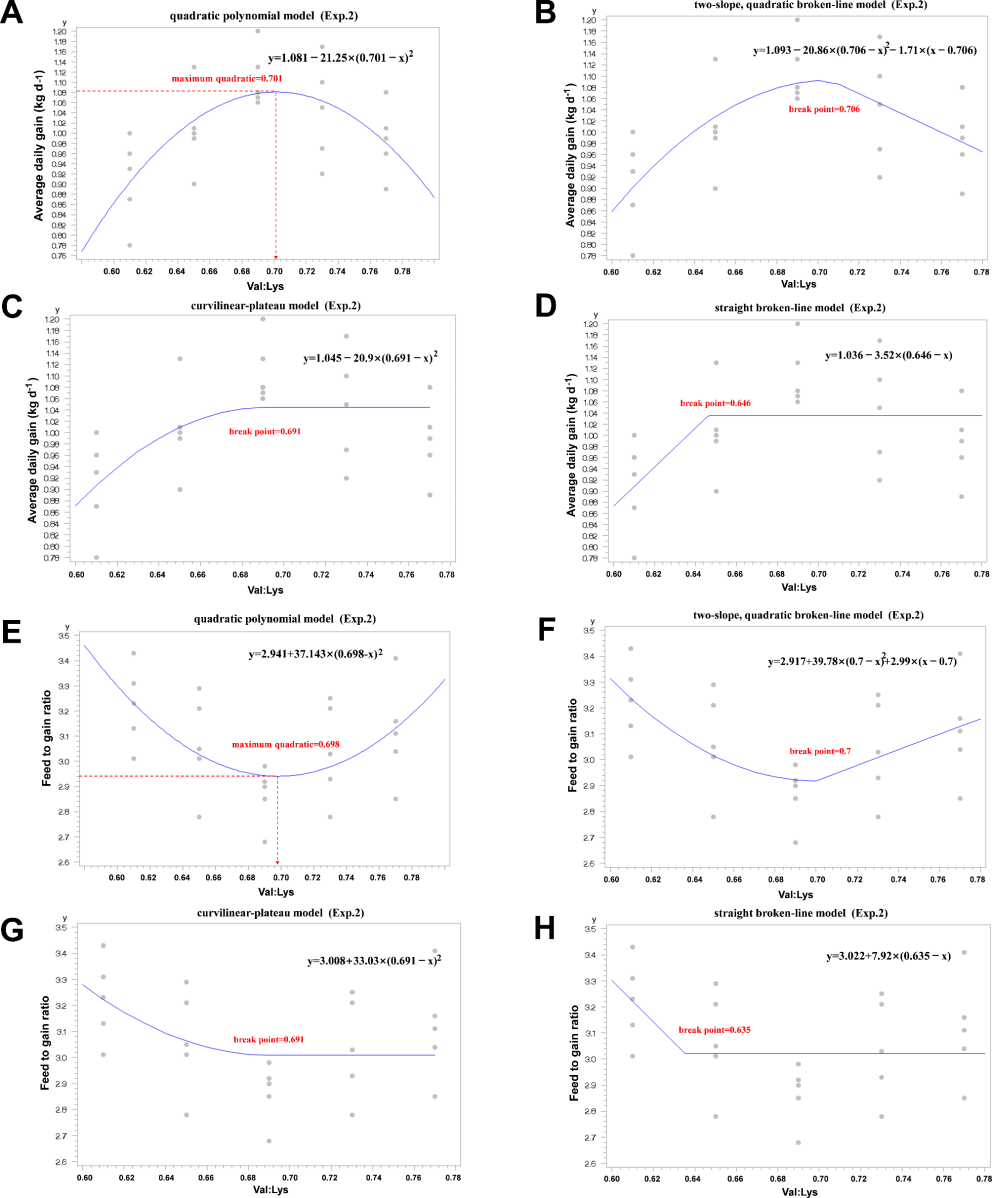
Estimation of dietary SID Val: Lys ratio for finishing pigs (Exp.2). Fitted plot (quadratic polynomial model, two-slope quadratic broken-line model, curvilinear-plateau model, and straight broken-line model) of ADG (**A-D**) and **F**: **G** (**E-H**) for pigs (75 to 100 kg bodyweight). The optimal Val requirement for ADG was 0.701, 0.706, 0.691, and 0.646, respectively (**A-D**). The optimal Val requirement for **F**: **G** was 0.698, 0.70, 0.691, and 0.635, respectively (**E-H**)

Finishing pigs (75 to 100 kg) consuming diets with 0.61 SID Val: Lys ratio had lower ADG compared to those fed 0.69 SID Val: Lys in diets (*P* < 0.05). Both ADG and F: G increased quadratically (*P* < 0.05) with increasing Val supply. Using regression models, estimate of the optimal SID Val: Lys ratio for ADG was 0.70 (quadratic polynomial model [R^2^ = 0.42]), 0.71 (two-slope quadratic broken-line model [R^2^ = 0.43]), 0.69 (curvilinear-plateau model [R^2^ = 0.30]), and 0.65 (straight broken-line model [R^2^ = 0.38]), respectively (Fig. 2A, B, C, and D). The optimum SID Val: Lys ratio predicted by quadratic polynomial model (R^2^ = 0.29, Fig. 2E) and two-slope quadratic broken-line model (R^2^ = 0.31, Fig. 2F) for F: G was both at 0.70, while curvilinear-plateau model (R^2^ = 0.18, Fig. 2G) and straight broken-line model (R^2^ = 0.20, Fig. 2H) estimated the Val requirement as 0.69 and 0.64, respectively.

### Experiment 3

As demonstrated in Table 4, no significant improvement was found for FBW, ADFI, ADG, or F: G of late finishing pigs from 100 to 130 kg, when SID Val: Lys ratio in diets increased from 0.61 to 0.77. Interestingly, from a numerical point of view, the highest ADG and the lowest F: G was observed in pigs receiving 0.73 dietary SID Val: Lys ratio. Val intake and Val: G increased linearly (*P* < 0.05) with dietary Val addition while there was no linear or quadratic improvement observed for ADG or F: G with increasing dietary SID Val: Lys ratio.

**Table 4:** Performance of pigs during late finishing periods (experiment 3)

	SID Val to Lys ratio						*P* value		
Item	0.61	0.65	0.69	0.73	0.77	SEM	ANOVA	Linear	Quadratic
IBW, kg	103	103	100	101	101	0.40	0.110	0.052	0.195
FBW, kg	128	129	127	129	127	0.51	0.507	0.507	0.871
ADFI, kg	3.34	3.31	3.32	3.33	3.32	0.00	0.165	0.294	0.218
Val intake, g	10.4^e^	11.0^d^	11.7^c^	12.4^b^	13.0^a^	0.22	0.000	0.000	0.410
Val:G	11.3	11.2	12.0	12.4	13.7	0.27	0.006	0.000	0.445
ADG, kg	0.92	0.99	0.98	1.01	0.96	0.01	0.407	0.317	0.130
F:G	3.64	3.36	3.41	3.32	3.48	0.05	0.360	0.329	0.108

Abbreviations: SID = standardized ileal digestible; IBW = initial bodyweight; FBW = final bodyweight; ADFI = average daily feed intake; Val intake = daily SID Val intake; Val: G = daily SID Val intake (g)/average daily gain (kg); ADG = average daily gain; F:G = feed to gain ratio; *n* = 4

## Discussion

The aim of this study was to evaluate the dietary SID Val: Lys requirement in finishing pigs for optimal performance under commercial conditions. In the present study, a basal SID Val: Lys ratio of 0.61 was used, with Lys being the second limiting thereafter. For 40 to 75 kg early finishing pigs in Exp.1, improved FBW, ADG and F: G was found in pigs receiving 0.65 or 0.69 dietary SID Val: Lys ratio while no improvement in ADFI was obtained with increasing Val supply. In line with a previous study [5], growth performance of pigs received a basal diet deficient in Val was significantly reduced in Exp.1, suggesting a growth retarding effect of Val deficiency. Val requirement was found to differ depending on the models and response criteria chosen. Based on quadratic analysis models, the estimated Val requirement for the early finishing pigs in Exp.1 was between 0.65 and 0.68. The estimates obtained by quadratic polynomial model and two-slope quadratic broken-line model were in accordance with a recent study showing that Val requirement for pigs between 39 and 68 kg of bodyweight was 0.68 [7], while lower than the quadratic requirement of 0.72 reported in another experiment [6]. Moreover, our estimate based on curvilinear-plateau model was in line with the NRC (2012) [9] recommendation of 0.65 for 40 to 75 kg pigs, while estimates obtained from straight broken-line models were lower than recommendation.

For 75 to 100 kg finishing pigs in Exp.2, pigs consuming 0.61 SID Val: Lys in diets had lower ADFI compared to those fed 0.69 or 0.73 SID Val: Lys in diets. These findings were in agreement with a previous report which showed that Val deficiency was associated with decreased weight gain and feed intake [5]. Nakahara et al. [12] revealed that ingestion of a Val deficient diet resulted in a remarkable reduction of food intake and hypothalamic somatostatin may be a central mechanism of the anorexia induced by Val deficiency. Given that no significant change in ADFI was observed in Exp.1 or Exp.3, it remained unclear why the Val requirement for ADFI was not responding in a dose-dependent manner. In Exp.2, the optimal SID Val: Lys ratio acquired by three quadratic regression models was between 0.69 and 0.71, when using ADG and F: G as response criteria. Noticeably, these estimates were higher than NRC (2012) [9] recommendation of 0.66 for 75 to 100 kg pigs. In contrast, our estimates based on straight broken-line models were lower than recommendation. One possible explanation for this discrepancy may be that the estimates at 100% of the maximum response of quadratic models usually appeared to overestimate the nutritional need for pigs, while a broken-line model might underestimate the requirement [6, 13]. In a dose-response study in 71 to 92 kg finishing pigs, Liu et al. [6] revealed that the optimal SID Val: Lys for ADG was determined to be 0.72 using a quadratic model, which was higher than that of the presented study.

For late finishing pigs from 100 to 130 kg in Exp.3, although no significant improvement for performance were found, 0.73 SID Val: Lys in diets resulted in the highest FBW and ADG while the lowest F: G from a numerical point of view. The absence of statistical significance in Exp.3 may result from the BCAA interactions. For late finishing pigs in Exp.3, the dietary crude protein content was set at a very low level (8.87%). Due to higher corn inclusion, which contained greater levels of Leu, it was not easy to formulate the SID Leu: Lys ratio in corn-soybean meal diets at the level recommended by NRC (2012) [9]. The SID Leu: Lys ratio was about 1.48 in Exp.3, which was in great excess for Leu. An excess of dietary Leu may enhance the catabolism and oxidation of all BCAA and consequently increase the nutritional requirement for Val and Ile [14]. High level of Leu may also impair the performance and feed intake via stimulation of mTOR or indirect activation of GCN2 signaling pathway due to the reduced availability of Val [15]. Additionally, imbalanced Leu: Val supplementation may also influence the performance via affecting lipid metabolism and fat deposition [16]. Hence, the antagonistic effect of Leu might be more potent on Val for late finishing pigs in Exp.3, since this rearing phase is a critical timepoint for lipid accretion. Another possible reason for the lack of response might be the limited synthesis of non-essential AA for pigs due to the low level of crude protein intake [17]. These observations were consistent with prior research indicating that it was necessary to redefine Val requirement, since it might be underestimated in low-protein diets [16].

## Conclusions

Taken together, supplementation of Val to a Val-deficient basal diet improved growth performance in finishing pigs. The optimum SID Val: Lys ratio for pigs from 40 to 75 kg bodyweight was between 0.62 and 0.68, and for pigs from 75 to 100 kg was estimated to be between 0.64 and 0.71, using different regression models. Performance equations estimated in the current study may help nutritionists to determine Val levels in actual diet formulation with different production purposes.

## Data Availability

All data will be available from the corresponding author upon reasonable request.
